# Supplementary material for: Conditioner application improves bedding quality and bacterial composition with potential beneficial impacts for dairy cow’s health
Source: Microbiol Spectr. 2024 Feb 20;12(4):e04263-23. doi: 10.1128/spectrum.04263-23 (PMC10994720; doi:10.1128/spectrum.04263-23)
Supplement: Supplemental Figures S1 to S3 and supplemental Table S1 — Supplemental figures and tables. [file spectrum.04263-23-s0002.docx]

**Supplementary Figures and Tables**


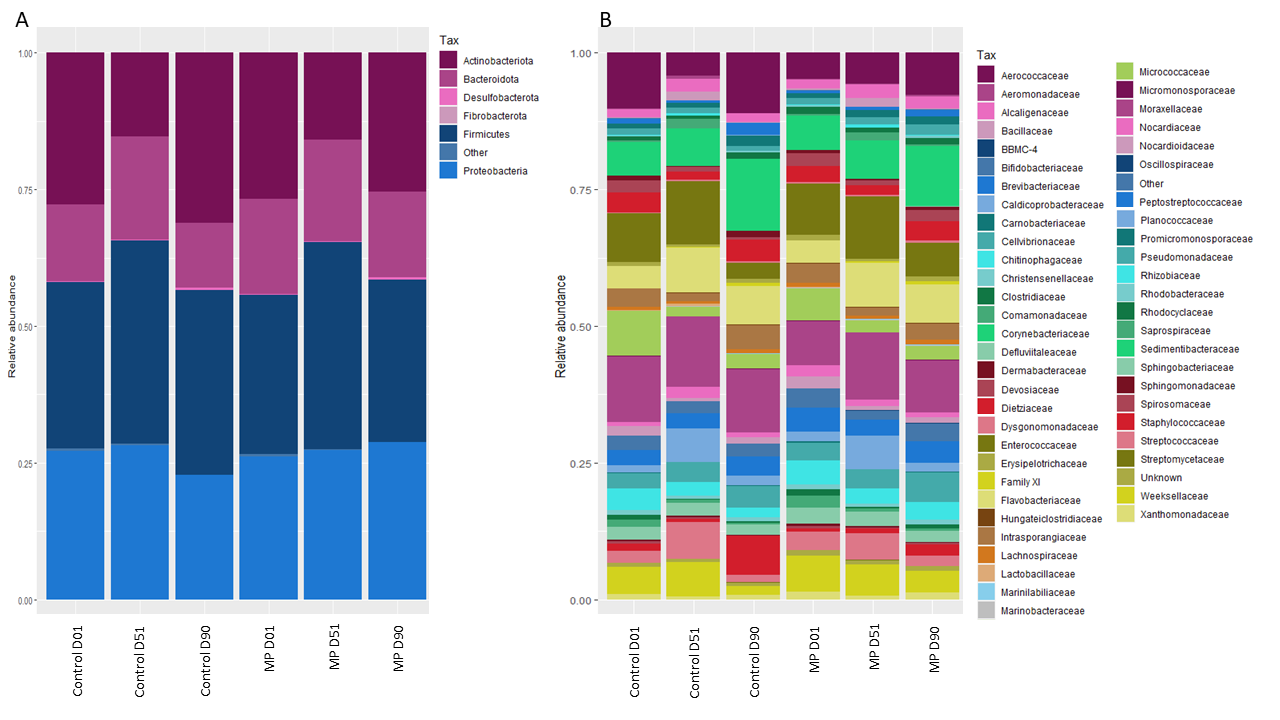


Supplementary Figure 1: Bacterial composition at phylum level (A) and Family level (B) of Control and ManurePro (MP) treated beddings (n=12) collected at D01, D51 and D90.


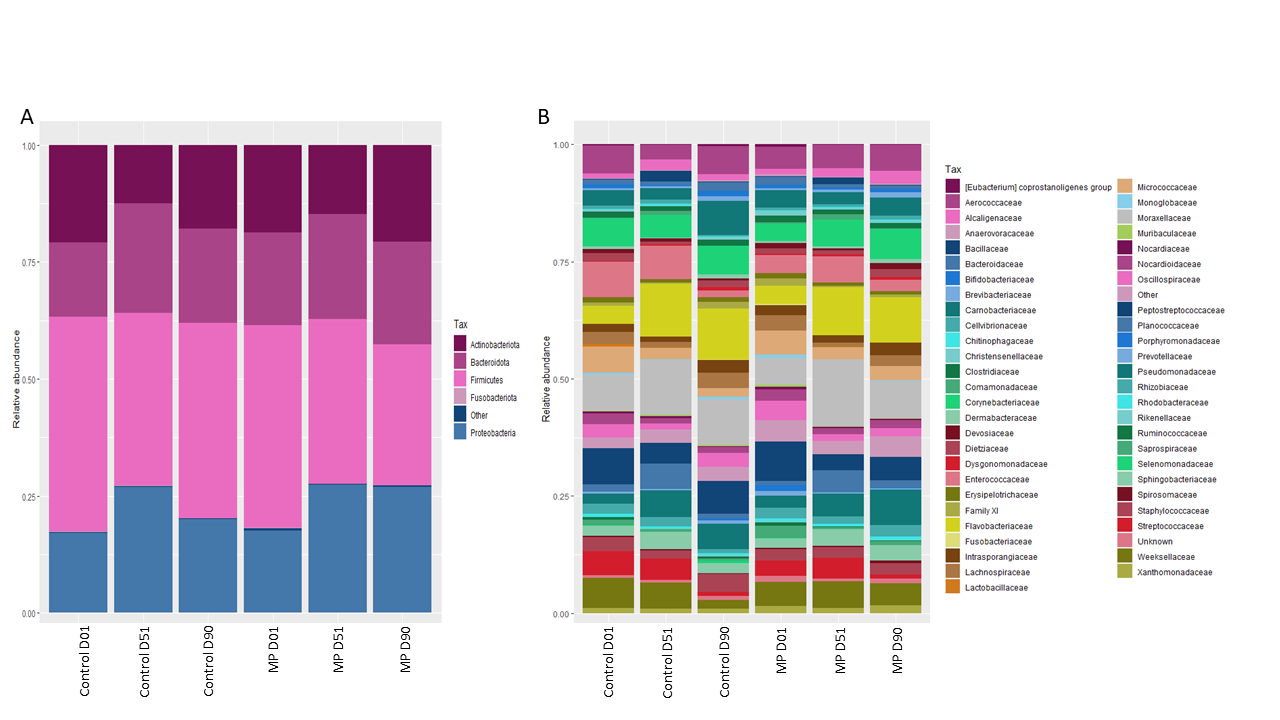


Supplementary Figure 2: Bacterial composition at A) Phylum and B) Family level of Control and ManurePro (MP) treated teat skin samples (n=18) collected at D01, D51 and D90.


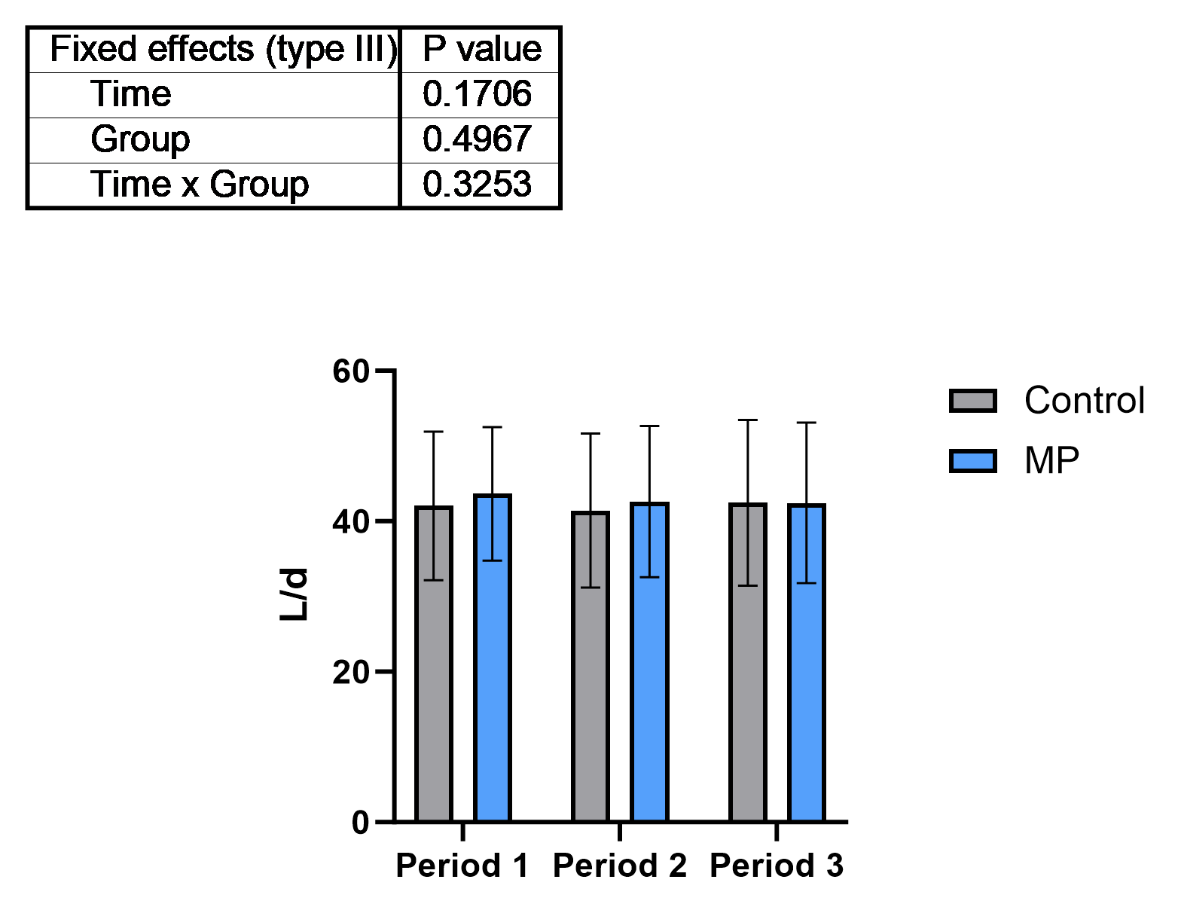


Supplementary Figure 3: Average milk yield (L/d) for cows in Control (grey, n=113) or ManurePro (MP, blue, n=115) groups in Period 1 (D01-D30), Period 2 (D31-D60) and Period 3 (D61-D90).

| **Mastitis detection (nb of cases)** | **Control** | **MP** | **P-value** |
| --- | --- | --- | --- |
| W1 | 6 | 10 | 0.438 |
| W2 | 5 | 5 | 0.999 |
| W3 | 11 | 11 | 0.999 |
| W4 | 9 | 8 | 0.806 |
| W5 | 8 | 13 | 0.36 |
| W6 | 8 | 17 | 0.089 |
| W7 | 10 | 12 | 0.823 |
| W8 | 11 | 7 | 0.336 |
| W9 | 6 | 6 | 0.999 |
| W10 | 7 | 7 | 0.999 |
| W11 | 11 | 8 | 0.482 |
| W12 | 7 | 11 | 0.463 |
| W13 | 5 | 9 | 0.409 |
| overall | 104 | 124 | 0.272 |

Supplementary Table 1: Number of suspicious mastitis cases through milk conductivity in Control (n=113) or ManurePro (MP, n=115) groups by week or over the whole experimental period and P-values associated.
